# Multifaceted role of clay minerals in pharmaceuticals

**DOI:** 10.4155/fso.15.6

**Published:** 2015-11-01

**Authors:** Inderpreet Singh Khurana, Satvinder Kaur, Harpreet Kaur, Rajneet Kaur Khurana

**Affiliations:** 1 Department of Pharmaceutical Sciences & Drug Research, Punjabi University, Patiala 147002, India; 2 GHG Khalsa College of Pharmacy, Gurusar Sadhar 141104, District Ludhiana, India; 3 Department of Botany, GHG Khalsa College, Gurusar Sadhar 141104, District Ludhiana, India; 4 University Institute of Pharmaceutical Sciences, Panjab University, Chandigarh 160014, India

**Keywords:** clay minerals, drug delivery, kaolinite, pharmaceuticals, phyllosilicates

## Abstract

The desirable physical and physiochemical properties of clay minerals have led them to play a substantial role in pharmaceutical formulations. Clay minerals like kaolin, smectite and palygorskite-sepiolite are among the world's most valuable industrial minerals and of considerable importance. The elemental features of clay minerals which caused them to be used in pharmaceutical formulations are high specific area, sorption capacity, favorable rheological properties, chemical inertness, swelling capacity, reactivity to acids and inconsiderable toxicity. Of course, these are highly cost effectual. This special report on clay minerals provides a bird's eye view of the chemical composition and structure of these minerals and their influence on the release properties of active medicinal agents. Endeavor has been made to rope in myriad applications depicting the wide acceptability of these clay minerals.

## Abstract (lay abstract)

With a variegated range of physical, chemical and physicochemical properties, clay minerals have an undisputable role in pharmaceutical industries. The minerals employed primarily are oxides, carbonates, sulphates, chlorides, hydroxides, sulphides, phosphates, nitrates, borates and phyllosilicates. These are widely put into use owing to their unique features. The significant contributions of these minerals are in therapeutic, cosmetics and as bulk forming agents. These are biologically compatible and hence ensure the genesis of safe and effective drug delivery systems.

**Figure 1.**
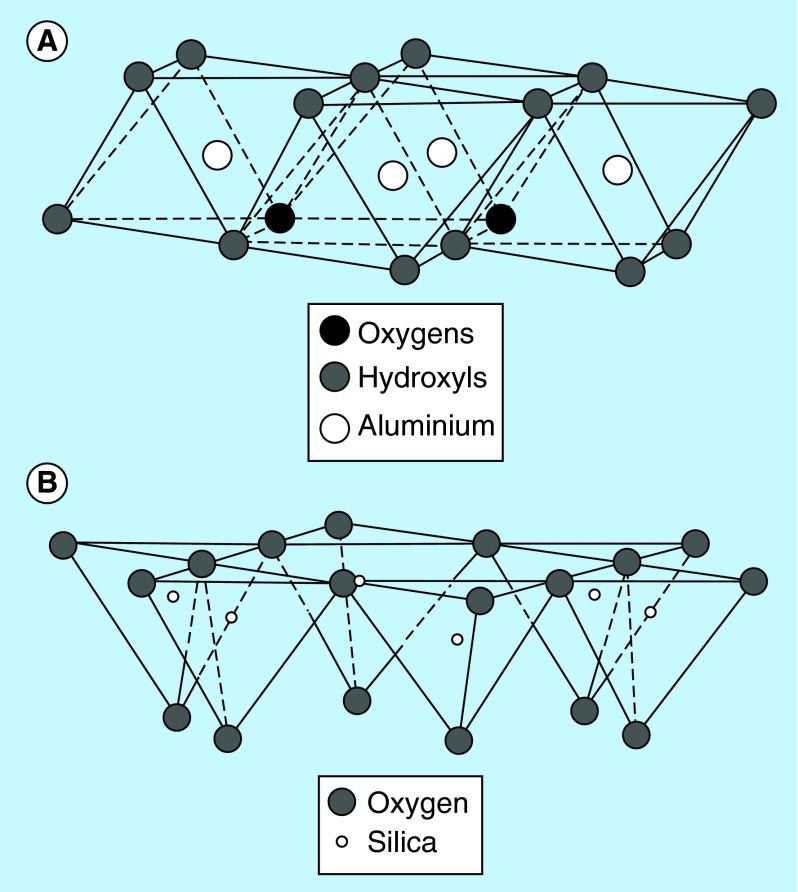
**Basic composition of clay minerals.** Diagrammatic representation of the **(A)** octahedral sheet and **(B)** tetrahedral sheet.

**Figure 2.**
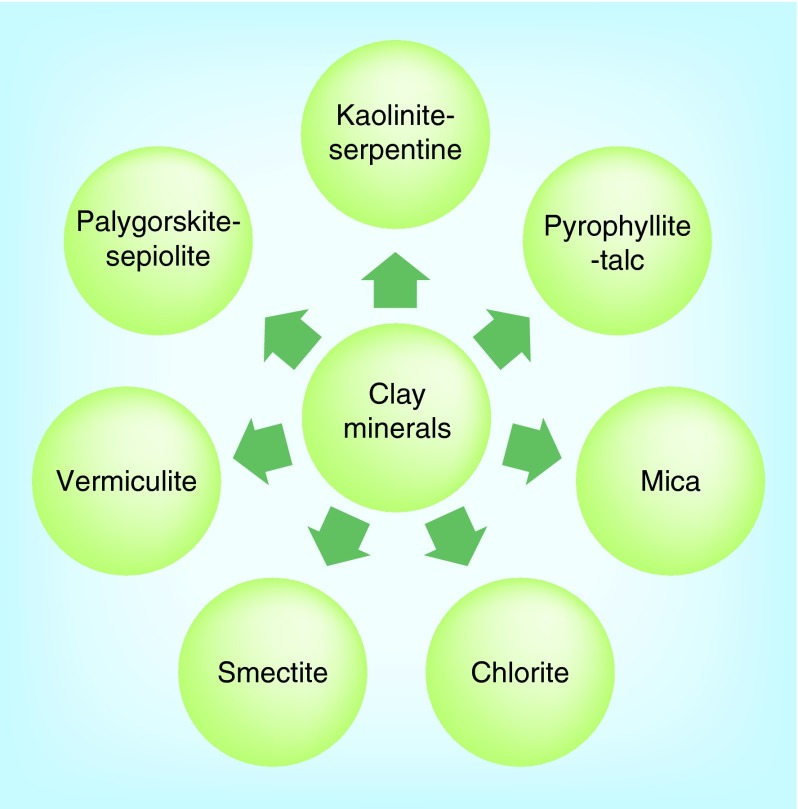
**Classification of clay minerals.**

Clay, a group of natural minerals with plastic properties are primarily composed of hydrous-layer silicates of aluminium, occasionally containing magnesium and iron particles of smaller size, in other words, less than 2 μm (7.9 × 10^−5^ inch). Hence, in broader terms, clay minerals practically involve minerals of the above-cited particles size. These are essentially composed of silica, alumina, magnesia, iron and water with varying degree of potassium, sodium and calcium [1].

Besides clay minerals no other minerals quite as immensely attract pharma people. Since the 19th century clay minerals have been explored on the geological, geotechnical and mineralogical fronts; their myriad therapeutic aspects were highlighted primarily in the pharmaceutical research. Clay minerals are a class of phyllosilicates which usually form as a result of chemical weathering of silicate minerals at the surface of the Earth [2]. Clay minerals are widely used in the pharmaceutical industry as lubricants, desiccants, disintegrants, diluents, binders, pigments and opacifiers. The other vital uses are as emulsifying, thickening, isotonic and anticaking agents. These also serve as flavor correctors and carriers of active ingredients. Other unique features are dispersivity, hygroscopicity, unctuosity, thixotropy and their rendering a slightly alkaline reaction as pH is slightly more than 7, plasticity, opacity and various high-quality colors [3].

## Composition & structural features of clays

Clay minerals are primarily of a fine-grained natural material with particle size <2 μm [4]. The physical and chemical properties of a particular clay mineral are dependent on its structure and composition. The structure and composition of the major industrial clays, in other words, kaolins, smectites and palygorskite–sepiolite, are very different even though each is comprised of octahedral and tetrahedral sheets as their basic building blocks. However, the arrangement and composition of the octahedral and tetrahedral sheets, as depicted in Figure 1, account for most differences in their physical and chemical properties [5]. Clay minerals are stacked, polymeric sandwiches of tetrahedral and octahedral sheet structures. They are classified first into ‘layer types,’ differentiated by the number of tetrahedral and octahedral sheets that have combined, and then into ‘groups,’ differentiated by the kinds of isomorphic cation substitution that have occurred [6,7]. Clay minerals can be classified into seven groups as illustrated in Figure 2. Thus, mineral products for pharmaceutical use vary according to composition, crystallinity, habit and texture, greatly affecting their properties [8]. The individual layers of clay minerals are composed of two, three or four sheets [9]. The sheets are formed either by tetrahedrons [SiO_4_]^4-^, abbreviated as ‘T’ or by octahedrons, for example, [AlO_3_(OH)_3_]^6-^, abbreviated as ‘O.’ The interior of tetrahedrons and octahedrons contain smaller metal cations, their apices being occupied by oxygen, which are with protons (as OH). All these fundamental structural elements are arranged to form a hexagonal network in each sheet. Based on the number and the ratio of sheets in a fundamental structural layer, the existing cation substitutions in the octahedrons and tetrahedrons and based on the resulting charge of the layers, the crystalline clay minerals are classified [10]. A detailed description of the chemical composition of the various clay minerals is given in Table 1.

Some predominantly employed clay minerals are kaolinite, 2SiO_2_·Al_2_O_3_·2H_2_O; pyrophyllite, 4SiO_2_·Al_2_O_3_·H_2_O; talc, 4SiO_2_·3MgO·H_2_O and chamosite, 3SiO_2_·Al_2_O_3_·5FeO·4H_2_O. The SiO_2_ ratio in a formula is the key factor determining clay mineral types [1].

## A sneak peek into the open-ended research on clay minerals

Clay minerals are used as excipients in pharmaceutical preparations to enhance their organoleptic characteristics, such as flavor (flavor correctors) and color (pigments), improve their physicochemical properties, such as viscosity of the active ingredients (emulsifying, thickening and anticaking agents), facilitate their elaboration (lubricants, diluents, binders, isotonic agents) or conservation (desiccants, opacifiers) and facilitate liberation of the active ingredient within the organism (disintegrants, carrier releasers) [3].

Clay minerals with very fine, thin particles and high adsorbent properties are quite useful for the antibiotics sorption. Kim *et al*. studied the sorption of oxytetracycline on clay minerals especially in acidic soils with high organic matter content [10]. The adsorption of four widely used drugs, carbamazepine, diclofenac, ibuprofen and ketoprofen, was investigated onto a porous silica under varied ionic strengths, and with different anions, divalent cations (Ca^2+^ and Mg^2+^), trivalent cations (Al^3+^ and Fe^3+^) and natural organic matter. The studies demonstrated that at a given pH the adsorption was most affected by ionic strength, trivalent cations and properties of pharmaceuticals. The increase of ionic strength resulted in an increase in the adsorption of ketoprofen, but a decrease in the adsorption of carbamazepine [11]. Cation exchange was the major mechanism of ciprofloxacin desorption from clay mineral surface. Ciprofloxacin desorption from kaolinite and montmorillonite was investigated under different pHs, different concentrations of metal cations of various valencies (Na^+^, Ca^2+^ and Al^3+^) and a cationic surfactant hexadecyl trimethylammonium (HDTMA), with different desorption cycles [12]. Enhanced desorption hysteresis of carbamazepine was observed for the smectites with negatively charged sites compensated with inorganic cations such as K^+^, Ca^2+^ and NH_4_^+^ than the desorption from organic cation-modified smectites (e.g., HDTMA clay), suggesting that the intercalated carbamazepine molecules are more resistant to release than carbamazepine partitioning in alkyl organic phase [2]. In addition, the large cation exchange capacity and surface area make the clay a good candidate to remove cationic pharmaceuticals from the effluent of waste water treatment facilities [13]. The protein adsorption capacity and selectivity of kaolinite and metakaolinite show a clear dependence on the chemical nature of the adsorbents surface and on the textural properties. Kaolinite and metakaolinite exhibit a very high affinity and good retention capacity for proteins like bovine serum albumin specially A-LA and B-LG [14]. The clay/poly(N-isopropylacrylamide) (PNIPAm) nanocomposite hydrogels, using lithium magnesium silicate hydrate as a clay mineral physical cross-linker were prepared to remove crystal violet from aqueous solution [15]. Similarly, Ballav and associates studied the absorption behavior of polypyrrole-coated halloysite nanotube nanocomposite [16]. Recently, clays have been modified through several approaches like conventional ion exchange reactions, sol-gel linking, atom transfer radical polymerization and polymer intercalation. The organic interaction incorporates different noncovalent bonding forces, such as amido acid five-membered ring chelation, carboxylic acid chelation, intermolecular hydrogen bonding and double-layer hydrophobic alignment in a layered clay confinement. Furthermore, the layered structure could be totally exfoliated and structurally randomized into individual silicate platelets using different mechanisms, such as the phase inversion of amphiphilic copolymer emulsifiers and phase transitions that involve zigzag Mannich polyamines. Different intercalation and exfoliation strategies help in developing detailed understanding of clay chemistry, thus exploring wider horizons of clay applications [17].

Clay minerals sorption activity is the most suitable application in veterinary science. Kaolins and smectites are most commonly used in animal nutrition as growth promoters and supplements for the treatment of gastrointestinal disturbances, particularly diarrhea [18].

The antibacterial activity of silver and cationic surfactant modified smectites from North Patagonia, Argentina, were tested in growth inhibition of *Escherichia coli* bacteria by the test of susceptibility on solid medium [19]. Minerals also enjoy diagnostic, odontological and traumatological applications, and are used in spas and aesthetic centers for therapeutic proposes [20]. Research focused on the role of clay minerals in kerogen formation, kerogen conversion to petroleum, oil migration and entrapment in reservoirs identify significant interactions arising from the adsorptive and catalytic properties of clay minerals and structural changes during diagenetic transformations [21]. Recently, Das *et al*., 2014 explored the significant antibacterial activity of copper nanoparticle-decorated organically with modified montmorillonite/epoxy nanocomposites against ubiquitous Gram- negative bacteria *Klebsiella pneumonia* and Gram-positive bacteria *Staphylococcus aureus* [22].

Kaolinite, talc, palygorskite and smectites are used for therapeutic purposes in pharmaceutical formulations as active principles or excipients. The possible use of sepiolite as active principle or excipient in pharmaceutical formulations was also investigated. Kaolinite, talc, palygorskite and smectites are used as excipients in cosmetics and pharmaceutical preparations [23]. A summary of the pharmacological activities of the clay minerals is described in Table 2. They also have an admirable role in the targeted and modified drug delivery system as in Table 3.

## Conclusion & future perspective

Research trends on clay minerals are heading toward the synthesis of minerals based on atomic and molecular scale design by affecting their physicochemical properties and thus they have a wide scope of applications in pharmaceutics. Chemical and physical interactions of clays with water and many other chemical species, and their dynamics, offer further scope. Biological implications related to clays are likely to be investigated more extensively. Novel materials based on nanotechnology, biochemical and medical applications, and environmental aspects are envisaged [55]. Chemically modified clay mineral electrodes are also being explored for many chemical sensor applications. Tuning the process and coupling it with a separation technique can achieve effective DNA quantification. Because of the stability of clays, combining them with enzymes and suitable redox mediators – for example, clay in conjunction with hydroquinone mediator – could be a new way to quantify microbiological systems such as fungi and bacteria such as *Escherichia coli* [56]. Novel-layered nanohybrid materials with controlled functions and microstructures are also being extensively explored. Nanocomposites based on clays and organic compounds are expanding. Studies of domain structures in layer silicates will continue, and further refinements in mixed-layer structure analysis can be expected. The study of clay mineral synthesis and alteration in nature shows signs of important reactivation. Furthermore, the self-assembled film of clay minerals has a highly regular multilayered nanostructure over a large area and could appreciably entrap in between the volume of air [57]. The combination of regular structure and substantial air volume contributes to the low thermal conductivity and flame blocking property of the film.

Clay-organic studies are developing in many directions. An understanding of the surface chemistry, particle shape and relative size distribution are crucial in developing such materials for an increasingly demanding and diverse world [58]. Organo-clays receive great interest for applications based on their capacity for selective adsorption of molecules. Thus, they have been used for application in chromatography separations, to remove organic pollutants from air and water, and to develop improved formulation for pesticides, as chemical sensor and molecular sieves, and so on. Among other properties applications based on special structural, gas barrier, antiflammability or others can be mentioned. Interesting photochemical behavior may also arise from the specific structure of those nanocomposites. Depending on the layer structure and specific properties, such as high-specific surface area, ion exchange capacity or hydration property, clay minerals are widely used in pharmaceuticals, and as adsorbents, catalysts or catalyst supports, ion exchangers and decolorizing agents.

**Table 1.** **Chemical composition of clay minerals.**

**Group**	**Chemical formulae**	**Octahedral character**	**Structure**
Kaolinite-serpentine	Al_2_Si_2_O_5_(OH)_4_	Trioctahedral dioctahedral ditriotahedral	Two-sheet phyllosilicates, where the T:O ratio = 1: 1 and the charge of the two-sheet layer = 0
Pyrophyllite-talc	Al_2_Si_4_O_10_(OH)_2_ Mg_3_Si_4_O_10_(OH)_2_	Trioctahedral dioctahedral	Nonswelling three-sheet phyllosilicates, where the T:O ratio = 2:1 and the charge of the three-sheet layer = 0
Smectite	Montmorillonite: (Al_1.67_Mg_0.33_)Si_4_O_10_(OH)_2_M^+^_0.33_	Trioctahedral dioctahedral	Strongly expanding three-sheet phyllosilicates, where the T:O ratio = 2:l and the charge of the three-sheet layer = 0.5–1.2
	Saponite: Mg_3_(Si_3.67_Al_0.33_)O_10_(OH)_2_M^+^_0.33_		
	Hectorite: (Mg,Li)_3_(Si,Al)_4_O_10_(OH)_2_M^+^_0.33_		
Vermiculite	(Mg,Fe,Al)_3_(Al,Si)_4_O_10_(OH)_2_·4H_2_O	Trioctahedral dioctahedral	The expanding three-sheet phyllosilicates, where the T:O ratio = 2:1 and the charge of the three-sheet layer = 1.2–1.8
Mica/Illite	KAl_2_(Si_3_Al)O_10_(OH)_2_	Trioctahedral dioctahedral trioctahedral	Three-sheet phyllosilicates, where the T:O ratio = 2:1 and the charge of the three-sheet layer ≤2
Chlorite	Al_4_[Si_8_O_20_](OH)_4_Al_4_( OH)_12_	Trioctahedral dioctahedral ditriotahedral	Four-sheet silicates, where the T:O:O ratio = 2:1:1 and the charge of the four-sheet layer is 1.1–3.3
Palygorskite- sepiolite group	(Mg,Al,Fe^3+^)_5_(Si,Al)_8_O_20_(OH)_2_(OH_2_)_4_.4H_2_O Mg_8_Si_12_O_30_(OH)_4_(OH_2_)_4_·8H_2_O	Trioctahedral dioctahedral	Palygorskite and sepiolite are phyllosilicates inas- much as they contain a continuous 2D tetrahedral sheet; however, they differ from other layer silicates in that they lack continuous octahedral sheets

**Table 2.** **Pharmaceutical activity of clay minerals.**

**Group**	**Pharmaceutical activity**	**Mechanism of action**	**Ref.**
Palygorskite-sepiolite, smectites	Gastric and duodenal ulcer	H^+^ neutralizing capacity decomposition in gastric acid and bring the bowel pH to 6	[24]
Kaolinite palygorskite-sepiolite, smectites	Gastrointestinal protector	High-specific area and sorption capacity	[25]
Palygorskite-sepiolite, smectites, kaolinite	Antidiarrhoeaics	Astringent action of the Ca^2+^ ion, which forms nonsoluble, hydrated phosphates	[26]
Kaolinite-talc, smectites	Dermatological protectors	Adhere to skin, forming a film that mechanically protects the skin. Adsorbs the skin's secretions, and creates a large surface for their evaporation which promotes a gentle antiseptic action by producing a water poor medium that is unfavorable for the development of bacteria	[27]
Mirabilite, epsomite, periclase brucite, magnesite	Laxatives	High solubility in water and HCl; release of Na^+^ or Mg^2+^ ions and nontoxic anions when ingested	[28]
Kaolinite	Anti-inflammatories and local anesthetics	High absorption and heat retention capacities	[19]
Palygorskite, sepiolite, kaolinite, smectites, talc	Cosmetic creams, powders and emulsions	Opacity and high sorption capacity	[29]
Silver and cationic surfactant-modified smectites	Antibacterial activity	Heavy metals modified montmorillonites exhibit high cation exchange capacity, large specific surface and colloid properties that give rise to optimum adsorbents of organic and inorganic substances	[30]
Halite, sylvite, melanterite, epsomite, mirabillite	Homeostatics	Smectite group of minerals have wider applications due to their high swelling and cation exchange capacity	[28]

**Table 3.** **Applications of clay minerals in drug delivery.**

**Type of drug-delivery system**	**Natural minerals employed**	**Mechanism**	**Ref.**
Extended release systems	Smectites montmorillonite fibrous minerals Hydrotalcite	They can retain large amounts of drug due to their high cation exchange capacity	[31–38]
Targeted delivery systems	Natural, synthetic, nanocomposites clay-polymers, films and hidrogels composites clay-polymers	Interact with drugs reducing their absorption. Therefore, such interactions can be used to achieve technological and biopharmaceutical advantages, regarding the control of release.	[39]
Colon delivery systems	Montmorillonite	Pharmaceutical natural minerals and drug interactions	[40,41]
Periodontal systems	Laminar minerals	Improved bioadhesion	[42,43]
Hydration-activated extended release systems	Smectites	Act as disintegrant agents in tablet formulations because of their hydrophilic and swelling properties	[44–46]
Microparticles	Amorphous silica bentonite attapulgite kaolin talc	Encapsulation of surface, precipitation inclusion and phamaceutical natural minerals–polymer interaction	[47–50]
Nanoparticles	Halloysite montmorillonites Bentonite porous silica	Pharmaceutical natural minerals provide spontaneous submicron dispersions in aqueous media, resulting in low cost and biocompatible systems with large surface area and high-inclusion capacity	[16,22,51]
Encapsulation of drugs inside layered double hydroxides (LDHs) with Mg^2+^, Al^3+^ and Fe^3+^ in the layers	Hydrotalcite	Hydrotalcite-layered solids with positively charged layers and charge-balancing anions in the interlayer space which protects drugs like nonsteroidal anti-inflammatory drugs in the GI tract	[52]
Cellular uptake	Hydrotalcite-derived antacidic and antipeptic formulations	Layered double hydroxides as nonviral vectors for delivery of antisense oligonucleotides	[53]
Silver nanoparticles and multiwalled carbon nanotubes	Montmorillonite	Transfection studies of these various functionalized nanopreparations implied that the gene delivery vector based on silver nanoparticles stabilized with starch and montmorillonite were more promising	[54]

**Executive summary**

**Unique features of clay minerals in pharmaceutical industry**

The enormous surface area, surface chemistry and surface charge impart significant and unique physical properties to the clay minerals, owing to which these possess tremendous scope to be utilized as therapeutic, cosmetics, functional, inert and bulk agents.
Most commonly employed clay minerals in pharmaceuticals and cosmetics are kaolinite, talc, montmorillonite, saponite, hectorite, palygorskite and sepiolite.
Precisely clay minerals serve as lubricants, desiccants, disintegrants, diluents, binders, pigments and opacifiers. The other imperative one are emulsifying, thickening, isotonic and anticaking agents.
The predominant curative properties include antacids, gastrointestinal protectors, antidiarrheaics, laxatives, homeostatics, emetics, antianemics and so on.
